# Discordant results between Xpert MTB/RIF assay and Bactec MGIT 960 culture system regarding the detection of rifampin-resistant *Mycobacterium tuberculosis* isolates in Wenzhou, China

**DOI:** 10.1128/spectrum.03859-23

**Published:** 2024-05-13

**Authors:** Guiqing He, Qingyong Zheng, Jing Wu, Lianpeng Wu, Zhi Geng, Guanglu Jiang, Hairong Huang, Xiangao Jiang, Xia Yu

**Affiliations:** 1 Department of Infectious Diseases, Wenzhou Central Hospital, The Dingli Clinical College of Wenzhou Medical University, The Second Affiliated Hospital of Shanghai University, Wenzhou, China; 2 Laboratory of Infectious Diseases, Wenzhou Central Hospital, The Dingli Clinical College of Wenzhou Medical University, The Second Affiliated Hospital of Shanghai University, Wenzhou, China; 3 National Clinical Laboratory on Tuberculosis, Beijing Key Laboratory on Drug-Resistant Tuberculosis, Beijing Chest Hospital, Capital Medical University, Beijing, China; 4 Department of Clinical Laboratory Medicine, Wenzhou Central Hospital, The Dingli Clinical College of Wenzhou Medical University, The Second Affiliated Hospital of Shanghai University, Wenzhou, China; 5 Beijing Synchrotron Radiation Facility, Institute of High Energy Physics, Chinese Academy of Sciences, Beijing, China; Quest Diagnostics Nichols Institute, Chantilly, Virginia, USA

**Keywords:** drug-resistant tuberculosis, borderline RIF resistance, rpoB mutations, Xpert MTB/RIF assay, Bactec MGIT 960

## Abstract

This study aimed to assess the possible causes of discordant results between Xpert MTB/RIF (Xpert) and Bactec MGIT 960 Culture System (MGIT960) regarding rifampicin (RIF) susceptibility in *Mycobacterium tuberculosis*. Patients with previous RIF-resistant tuberculosis who were admitted to Wenzhou Central Hospital from January 2020 to December 2022 were enrolled. The isolates obtained from these patients were subjected to RIF susceptibility tests using Xpert and MGIT960, and the minimum inhibitory concentration (MIC) of RIF was determined by the MYCOTB MIC plate test. Additionally, molecular docking and molecular dynamics (MD) simulations were performed to evaluate the binding efficacy of rpoB and RIF based on rpoB mutations detected in the isolates with discordant RIF susceptibility results. A total of 28 isolates with discordant RIF susceptibility test results were detected, 15 of them were RIF susceptible with MICs ≤ 0.5 µg/mL. Twelve out of 15 isolates contained borderline RIF resistance-associated mutations [L430P (*n* = 6), H445N (*n* = 6)], 1 isolate had D435Y and Q429H double mutation, and the remaining 2 isolates had a silent (Q432Q) mutation. Compared with the affinity of RIF toward the wild type (WT) (−45.83 kcal/mol) by MD, its affinity toward L452P (−55.52 kcal/mol), D435Y (−47.39 kcal/mol), L430P (approximately −69.72 kcal/mol), H445N (−49.53 kcal/mol), and Q429H (−55.67 kcal/mol) increased. Borderline RIF resistance-associated mutations were the main cause for the discordant RIF susceptibility results between Xpert and MGIT960, and the mechanisms of the resistance need further investigated.

**IMPORTANCE:**

This study is aimed at assessing discordant results between Xpert MTB/RIF (Xpert) assay and Bactec MGIT 960 Culture System (MGIT960) regarding the detection of rifampicin (RIF)-resistant *Mycobacterium tuberculosis* isolates in Wenzhou, China. The discordant results of RIF between these two assays were mainly caused by borderline RIF resistance-associated mutations, subsequently by silent mutations of rpoB. Borderline RIF resistance- associated mutations detected in our study were demonstrated to not be affected by the affinity of rpoB and RIF by molecular dynamics, and the mechanism of resistance was needed to be clarified. For the discordant results of RIF by Xpert and MGIT960 that occurred, *rpoB* DNA sequencing was recommended to investigate its association with resistance to RIF.

## INTRODUCTION

Tuberculosis (TB) is a serious public health concern, and in 2022, the World Health Organization (WHO) reported 10.6 million new cases of TB (1). Among the types of TB, the global incidence of multidrug-resistant TB (MDR-TB), which is characterized by resistance to isoniazid (INH) and rifampicin (RIF), was relatively stable from 2015 to 2022; however, it increased in 2022 with an estimated 0.41 million incident cases (1). Compared with drug-susceptible TB, MDR-TB is more difficult to treat and has fewer favorable outcomes (2, 3). Resistance to RIF is most commonly found in conjunction with resistance to INH, accounting for >85% of MDR-TB cases (4). Thus, developing rapid and accurate diagnostic methods or improving the performance of the existing methods is imperative to determine resistance to RIF.

Until now, several phenotypical and molecular assay techniques have been used to determine resistance to RIF (5). Phenotypical assays have been performed using the Bactec MGIT 960 Culture System (MGIT960) to determine susceptibility to RIF. Usually, culture-based methods are considered the gold standard for drug susceptibility testing (DST); however, molecular methods that use Xpert MTB/RIF (Xpert) provide rapid information about rpoB mutations associated with resistance to RIF. Thus, in a study, many individuals were studied prospectively to determine the incidence of discordant RIF susceptibility results between Xpert and MGIT960 (4, 6, 7), most of which were presented as RIF resistant by Xpert but susceptible by MGIT960. A high critical concentration of RIF and low bacterial load in specimens were considered the main reasons behind this discordance; thus, *rpoB* DNA sequencing was suggested to determine the molecular reason. However, the association between rpoB mutations and resistance to RIF remained unclear (8).

Herein, we aimed to assess *Mycobacterium tuberculosis* (MTB) isolates with discordant RIF susceptibility results acquired prospectively using Xpert and MGIT960. Next, the whole genome sequencing (WGS) and minimum inhibitory concentration (MIC) findings were analyzed to identify parameters implicated in conflicting RIF susceptibility results. Additionally, molecular docking and molecular dynamics (MD) analyses were performed to analyze rpoB mutations related to these discordant results in order to determine their effect on RIF binding affinity.

## MATERIALS AND METHODS

### Study design

The study was conducted from January 2020 to December 2022. We enrolled MTB-positive cases determined by Xpert from TB patients with previous RIF resistance and then performed MGIT960 culture. For the MTB-positive culture, DST of RIF was carried out by MGIT960. RIF resistance was determined by Xpert or MGIT960 using the baseline sputum.

### Smear and culture

Direct smear was prepared, and acid-fast bacilli was detected by Ziehl Neelsen carbol fuchsin smear microscopy. After processing with NALC/NaOH and centrifugation, the resuspended sputum pellet was subjected to culture in a liquid medium using the MGIT960 (BD Diagnostic Systems, New Jersey, USA). For all the isolates, MPT64 antigen testing was performed to confirm the presence of *M. tuberculosis* complex.

### DST by MGIT960

If the culture is worked up 1 or 2 days after signaling positive, it can be used directly to inoculate the DST MGIT tubes. If the culture is used to set up DST between 3 and 5 days after signaling positive, dilute 1 mL of MGIT broth in 4 mL of sterile saline (1:5 dilution). Then, the phenotypic DST of RIF was performed as per the manufacturer’s instructions. The critical concentration (CC) of RIF by MGIT960 was 1.0 μg/mL as defined by the manufacturer at the time of study. Then, the phenotypic DST of RIF was performed as per the manufacturer’s instructions.

### Minimum inhibitory concentration

The MYCOTB MIC plate test was performed per the manufacturer’s instructions to determine MICs (Trek Diagnostic Systems, Cleveland, OH, USA). Briefly, isolate suspensions were prepared using a solid culture and adjusted to the 0.5 McFarland standard. Next, 100 µL of the suspension was transferred into a tube containing 10 mL of 7H9 broth supplemented with oleic acid albumin dextrose catalase to yield 10^5^ colony-forming units/mL. Then, a 100-µL aliquot of the final inoculum was transferred into each well and the plate was covered with an adhesive seal and incubated at 37°C. The DST results were interpreted twice for each test, once when the drug-free control showed visible cell growth and then at the longest incubation time (21 days), as suggested by the manufacturer. MICs were recorded as the lowest antibiotic concentrations that reduced visible growth, and resistance to RIF on MYCOTB was defined as visible growth at or above 0.5 µg/mL (9). H37Rv [American Type Culture Collection (ATCC) 27294] and H37Ra (ATCC 25177) strains were used as controls.

### Xpert MTB/RIF

The Xpert assay was performed per the manufacturer’s instructions. Briefly, 1 mL of the sputum specimen was mixed with 2 mL of the sample reagent, vortexed for at least 10 s, and incubated at room temperature (25°C) for 15 min. Then, 2 mL of the resulting mixture was transferred into the cartridge, loaded into GeneXpert (Cepheid Inc., Sunnyvale, CA, USA), and the automatic detection procedure was run. For an invalid result, a repeat Xpert test was performed on the same sample.

### Whole genome sequencing

MTB DNA was extracted using a nucleic acid extraction kit (magnetic bead method), followed by library construction using a library construction kit. Next, library fragment sorting was performed. Agilent 2100 was used for quality inspection, and PE150 mode was used for sequencing using Illumina NovaSeq 6000.

The raw data were filtered to exclude adapters and low-quality bases using trimgalore (https://github.com/FelixKrueger/TrimGalore). Bowtie2 was used to map clean data onto the host genome to remove the host’s reads. Further, these data without the host’s reads were mapped against the pan-susceptible H37Rv reference genome to obtain MTB isolate sequences. Single nucleotide polymorphism calling was detected using snippy (https://github.com/tseemann/snippy).

### Molecular docking

Except for high confidence resistance-associated mutations, five rpoB mutations detected in discordant RIF-susceptible results were prepared by directly mutating corresponding amino acid residues using PyMOL, including moderate confidence-resistant mutation D435Y, borderline RIF resistance-associated mutations (L452P, L430P, and H445N), and uncertain resistant mutation Q429H. Molecular docking simulations of RIF against the WT and mutated rpoB models (PDB code: 5UHC) were performed using Autodock Vina 1.1.2 (10). Before docking, the receptor and ligand structures were prepared using AutoDockTools 1.5.7 (11). For receptor preparation, we deleted all water atoms and added hydrogen atoms to the structure. The Gasteiger charge model was further used to add partial charges to the receptor, and the final structure was saved as a pdbqt file for docking. For ligand preparation, we checked total charges on the residues, detected the torsion tree root, and assigned bond torsion as either rotatable or non-rotatable, and the final structure was saved as a pdbqt file.

Docking was performed with a default exhaustiveness of 50, which specifies the number of runs that start with a random ligand conformation, and a default of *n* poses of 9, which defines the final number of ligand poses to report. For more accurate docking results, a proper search box centered at the potential active site was defined. To this end, the position of RIF in the 5UHC PDB structure was defined as the center of the cubic box with a grid size of 40 × 40 × 40 Å. To further improve docking prediction quality, we repeated the molecular docking procedure twice. The predicted ligand pose with the lowest binding affinity was selected as the final docking model for further analysis. The receptor–ligand interactions were analyzed using LigPlot+ (12).

### MD simulations and analysis

The best-docked rpoB–RIF complex with the lowest binding affinity was selected for further MD simulations for each mutant and WT. Before the simulation, rpoB and RIF structures were processed to generate necessary topologies, and AmberTools23 (13) was used to generate RIF topology. First, AM1-BCC charges (14) and the GAFF2 force field (15) were added to RIF using the antechamber program (16). Next, topology and coordinate files in the AMBER format were generated using the tleap program. Finally, an RIF topology file in the GROMACS format was generated using ACPYPE (17). However, some atoms in the rpoB structure were missing; thus, the protein structure was preprocessed by automatically adding missing side-chain atoms before generating structural topology using COOT (18).

The MD simulations of the rpoB–RIF complex were performed using graphical processor unit (GPU)-enabled GROMACS 2019.6 (19). Additionally, the AMBER FF14SB force field (20) was used. Each system was solvated in a cubic box of water molecules extending at least 12 Å from the edge of the complex, and the TIP3P water model was used for simulation. Sodium chloride ions were added to neutralize the systems to a salt concentration of 150 mM. Approximately, each system comprised about 250,000 particles (~190,000 water molecules, ~700 sodium ions, ~600 chloride ions, and ~50,000 protein atoms) that were parameterized with the Amber14SB_parmbsc1 force field. For each system, the steepest descent algorithm was applied to minimize the system energy up to a gradient limit of 1,000 kJ/mol/nm. After energy minimization, each system was equilibrated in a canonical ensemble (NVT) at 300 K for 100 ps, followed by further equilibration in an isothermal–isobaric ensemble (NPT) at 1 Bar of pressure for 100 ps. During system equilibration, positional restraints were applied on non-hydrogen atoms of the complex with a restraint force of 1,000 kJ/mol/nm^2^ to avoid structural dissociation. Temperature and pressure coupling at 0.1 and 2.0 ps time constants were managed using V-rescale (21) and Parrinello–Rahman methods (22), respectively. Finally, production simulations were performed for each system under the NPT conditions with the harmonic restraints removed for 50 ns while applying periodic boundary conditions. For all simulations, the leap-frog algorithm was used as an integrator. Constraints were applied to hydrogen bonds (H-bonds) using the LINCS method (23). Long-range electrostatic interactions were applied using the particle mesh Ewald method (24) with a real-space cutoff of 14 Å. The cutoff radius for coulomb and van der Waals interactions was set to 14 Å. The integration time step of 2 fs was performed, and trajectory snapshots were recorded every 20 ps.

The analyses were performed with built-in tools in GROMACS, such as rmsd, rmsf, gyration, energy, hbond, and sasa to assess system properties, including the overall stability, local residue, and general structure fluctuations. H-Bonds between the rpoB protein and the RIF ligand were analyzed based on the following criteria: D–A distance cutoff = 3.5 Å and D–H–A angle cutoff = 30°, where D, A, and H were donor, acceptor, and hydrogen atoms linked to the donor atom. The binding free energy computations between *rpoB* and RIF were performed using trajectory snapshots ranging from 45 ns to 50 ns by following the molecular mechanics Poisson–Boltzmann surface area method using the gmx_MMPBSA toolbox (25). Specifically, the binding free energy (∆G_bind_) comprised electrostatic (∆E_ele_), van der Waals (∆E_vdw_), polar solvation (∆G_GB_), and non-polar solvation energies (∆G_sur_). UCSF Chimera (26) was used to visualize simulation trajectories, and PyMOL was used to prepare structural figures.

## RESULTS

### Discordant RIF susceptibility test results

**Fig 1:**
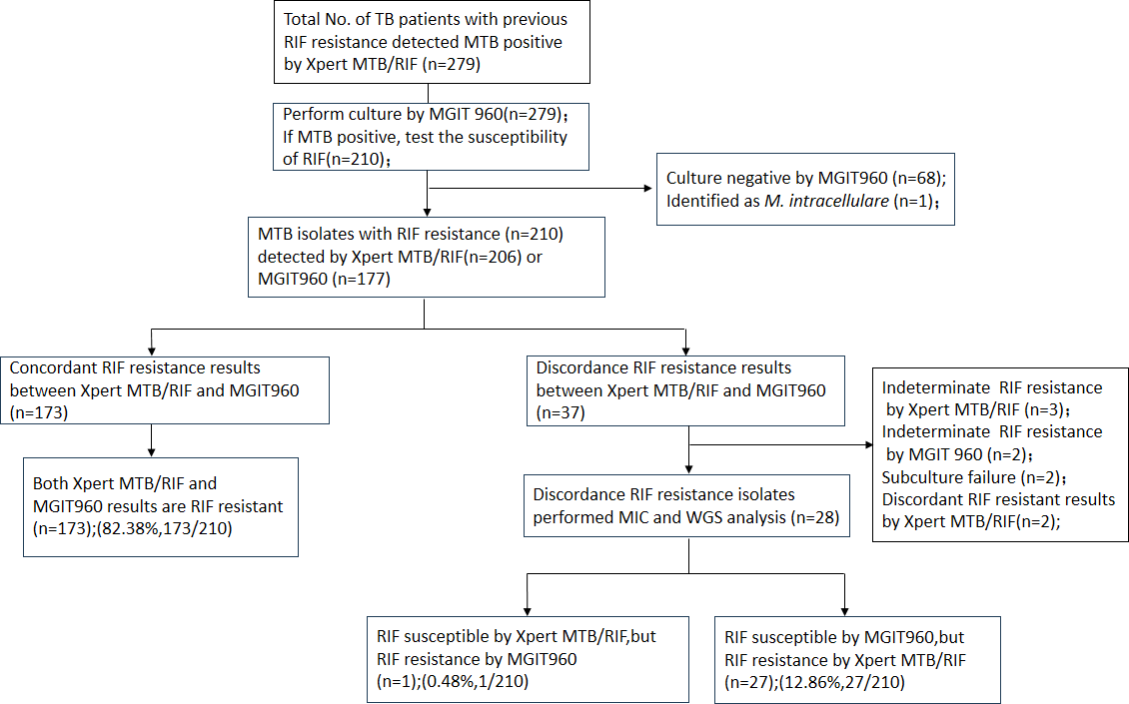
Overall study outline and result summary of Xpert assay, culture, and *rpoB* gene sequencing.

A total of 279 TB patients with previous RIF resistance underwent Xpert MTB/RIF with positive results which were included at Wenzhou Central Hospital between January 2020 and December 2022. Then, MGIT960 culture was performed for 279 included specimens. Among them, 68 patients were excluded from phenotypic DST owing to negative MGIT960 results, and one patient’s isolate was determined to be *M. intracellulare* by species identification. For the 210 MTB culture-positive isolates, 177 isolates were determined to be RIF resistant by MGIT960, and 206 isolates presented to be RIF resistant by Xpert MTB/RIF. Additionally, 173 isolates showed consistent RIF susceptibility results, whereas 37 isolates showed discordant RIF susceptibility results. Finally, 28 isolates with discordant RIF susceptibility results were further evaluated to clarify the discordance (Fig. 1).

### MICs and rpoB mutations in discordant RIF susceptibility test results

**Fig 2:**
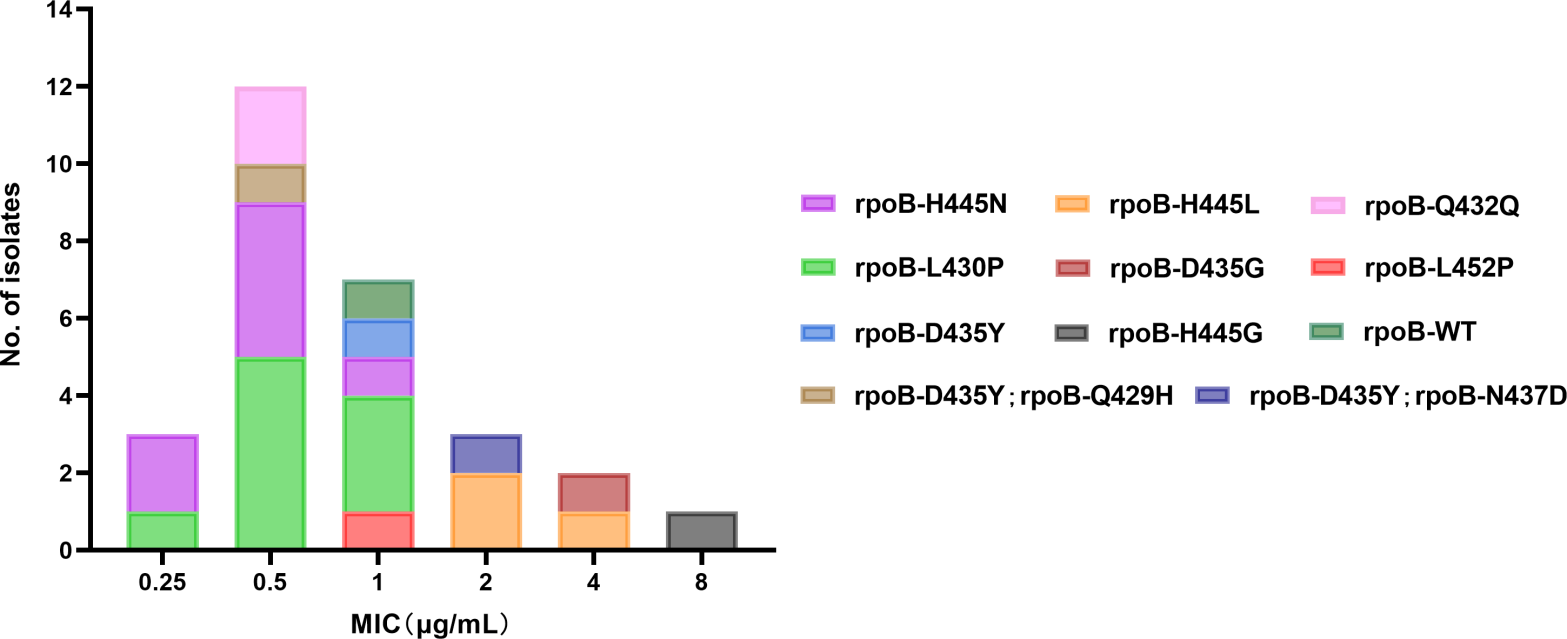
The MIC distributions grouped according to *rpoB* mutation profile among 28 isolates with discordant RIF-susceptible results.

A total of 28 isolates with discordant RIF susceptibility were detected; 27 of them were RIF resistant as shown by Xpert and RIF susceptible as shown by MGIT960, and only one isolate showed the opposite results. Further, we analyzed the distribution of MICs of these isolates to determine their associations with various rpoB mutations and resistance to RIF. Six isolates had MICs > 1 µg/mL, 7 isolates had MICs = 1 µg/mL, and the remaining 15 isolates presented MICs ≤ 0.5 µg/mL. Among six isolates with MICs > 1 µg/mL, five isolates owned high confidence-resistant mutations, including H445L (*n* = 3), H445G (*n* = 1), and D435G (*n* = 1). The remaining one isolate had D435Y and N437D double mutation. The discordant RIF susceptibility results among the six isolates were mainly attributed to false RIF susceptible by MGIT960. For the 15 isolates with MIC ≤ 0.5 µg/mL, 12 of them contained borderline RIF resistance-associated mutations [L430P (*n* = 6) and H445N (*n* = 6)], 1 isolate had D435Y and Q429H double mutation, and the remaining 2 isolates had a silent (Q432Q) mutation. Notably, no overlap was observed among rpoB mutations between RIF-susceptible and resistant isolates (Fig. 2). Furthermore, an isolate referred to as RIF resistant according to MGIT960 and susceptible according to Xpert results showed an MIC of 1 µg/mL with a silent mutation rpoB A1075A. However, no *rpoB* or *rpoC* non-synonymous mutations or heterogeneous resistance was detected by WGS.

### Molecular docking of mutant protein structures in discordant RIF susceptibility test

**Fig 3:**
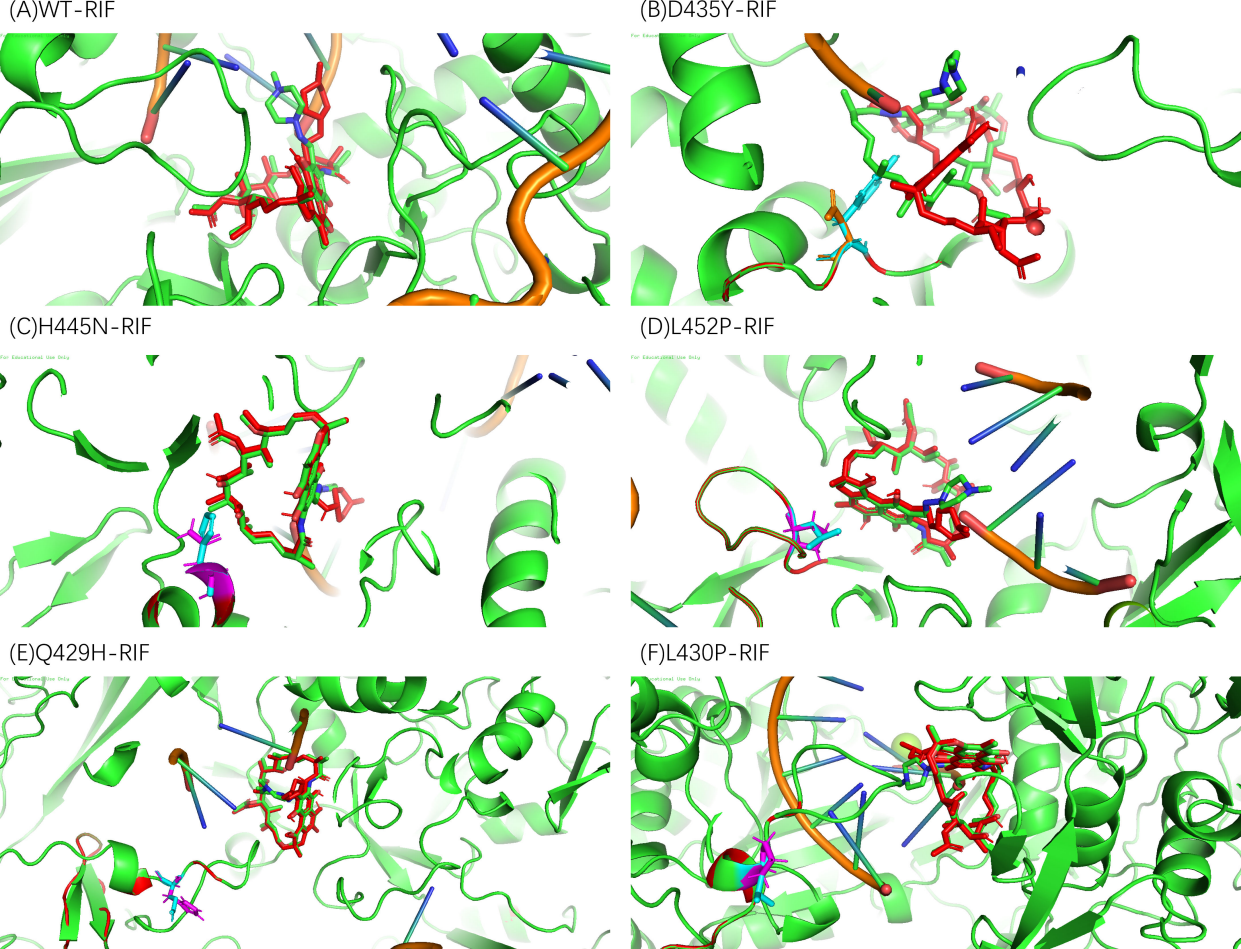
Post-docking analysis: the three-dimensional illustration of molecular interactions of RIF with the WT (**A**) and mutated forms of *Mtb* rpoB (**B–F**). RIF is shown as green sticks and red sticks after docking. Interacting atoms and associated residues are shown as cartoon (colored green). Amino acid substitutions are distinguished with different colors. (**B**) D435 colored by orange and Y435 colored by blue. (**C**) H445 colored by blue and N445 colored by purple. (**D**) L452 colored by blue and P452 colored by purple. (**E**) Q429 colored by blue and H429 colored by purple. (**F**) L430 colored by blue and P430 colored by purple.

In terms of docking scores and distances corresponding to those bonds, the post-docking analysis indicated various binding patterns of RIF with the WT and mutant models; 2D binding modes of RIF with the WT and mutated forms of *Mtb rpoB* were presented in Fig. S1. The mutant complexes mostly showed similar binding free energy values to those shown by the WT rpoB–RIF complex (−13.0 kcal/mol) except the rpoB D435Y–RIF complex (−9.8 kcal/mol), irrespective of where they were located within the RIF binding pocket (Table S1). Compared with the WT rpoB–RIF complex (Fig. 3A), the D435Y–RIF complex showed significantly higher ligand root-mean-square deviation scores (ligands in the mutant model compared with ligands in the WT model) and subpar docking scores. Nevertheless, none of the tested mutations were unable to bind with RIF (Fig. 3B through F).

### Effect of mutations on protein–ligand binding affinity by MD

Only one (D435Y) out of the five mutated rpoB models contained mutants with unfavorable protein–ligand interactions. To elucidate the effect of mutations on the dynamic behavior of RIF binding, 50-ns MD simulation experiments were performed on the WT and five models in the complex. Intermolecular H-bond numbers play a key role in stabilizing protein–ligand complexes. A more stable time period of the last 10 ns of MD was selected to calculate H-bond numbers and was compared with those of the WT (2.07 ± 0.997 H-bonds), D435Y (3.41 ± 1.68 H-bonds), H445N (2.1 ± 0.72 H-bonds), L452P (3.28 ± 0.68H-bonds), Q429H (4.8 ± 1.5 H-bonds), and L430P (3.75 ± 0.88 H-bonds), which generally recorded higher intermolecular H-bond numbers on average. These results suggested that the tested mutations did not destabilize RIF–rpoB interactions.

Single point mutations in ligand binding sites affect ligand binding thermodynamics. Usually, van der Waals interactions (ΔE_vdW_) are the driving force for RIF–rpoB interactions, whereas polar energy (ΔG_binding_) disturbs the binding. Compared with the binding affinity of RIF toward the WT (approximately −45.83 kcal/mol), its binding affinity toward D435Y (approximately −47.39 kcal/mol), H445N (approximately −49.53 kcal/mol), L452P (approximately −55.52 kcal/mol), Q429H (approximately −55.67 kcal/mol), and L430P (approximately −69.72 kcal/mol) increased (Table 1).

**TABLE 1:** Summary of binding free energy values acquired for RIF binds to *Mtb*-resistant mutation sites

Systems	ΔE_vdW_	ΔE_ele_	ΔG_solv_, polar	ΔG_solv_, non-polar	ΔG_gas_	ΔG_solv_	ΔG_binding_ (kcal/mol)
WT-RIF	−81.14	−37.51	82.69	−9.87	−118.65	72.83	−45.83
D435Y-RIF	−68.02	−72.47	102.31	−9.21	−140.49	93.11	−47.39
H445N-RIF	−75.46	−38.73	74.00	−9.35	−114.19	64.65	−49.53
L452P-RIF	−77.80	−60.02	91.96	−9.66	−137.82	82.30	−55.52
Q429H-RIF	−74.64	−64.52	93.32	−9.83	−139.16	83.49	−55.67
L430P-RIF	−81.21	−53.90	75.14	−9.76	−135.11	65.39	−69.72

## DISCUSSION

RIF has been considered a cornerstone of TB treatment, and the accuracy of RIF susceptibility results is pivotal to treatment efficacy. More than 95% of *MTB* clinical strains resistant to RIF harbors mutations in the 81-bp region of *rpoB*, known as the RIF resistance-determining region. The region spans codons 433–458, which is a hotspot for molecular diagnostic techniques to determine resistance to RIF. Several molecular diagnostic techniques, such as Xpert, can rapidly determine *MTB* resistance toward therapeutic drugs. Subsequently, a series of studies have shown that the rate of discordance between Xpert and MGIT960 with respect to detecting resistance to RIF ranged from 16.2% to 55.1% (5, 6, 27). There were several reasons behind this discordance: (i) these molecular methods might have missed isolates with resistance mutations occurring outside the rifampicin resistance-determining region (RRDR) region, such as rpoB Val170Phe and Ile491Phe (28), (ii) low bacillary loads may cause false RIF-resistant results by Xpert (29), and (iii) synonymous mutations in RRDR can also lead to false resistance results with Xpert (30), such as rpoB Q432Q silent mutations in our study and previous reported rpoB P514P (31).

A WHO report has revealed that disputed rpoB mutations are clinically relevant and should be called “borderline.” Thus, the six mutations (L430P, D435Y, H445L, H445N, H445S, and L452P) were considered borderline RIF resistance-associated mutations by the WHO, which showed a sensitivity of 92.3% (95% CI, 91.8%–92.8%) for predicting phenotypic DST for RIF susceptibility (32). Thus, borderline rpoB *m*utations are the main cause of discordant RIF susceptibility results, i.e., Xpert-resistant/MGIT960-susceptible results (33). However, the association of these borderline mutations and RIF-containing regimens remains unclear.

*In vitro* susceptibility tests showed the dispersed MIC distributions of the *MTB* isolates with borderline mutations in RIF-susceptible and resistant strains. Xia H et al. showed the MIC range of three borderline mutations containing the breakpoint of RIF (0.25 – >2 for rpoB L452P and H445N and 0.12 – >2 for rpoB L430P) (34). Li M et al. showed that rpoB D435Y was detected in an isolate with MICs of 0.5 µg/mL (35). We found substitutions in borderline RIF resistance-associated mutations L430P (*n* = 6) and H445N (*n* = 6), which showed all MICs below 0.5 µg/mL, although WHO lowered the RIF CC of MGIT960 from 1 to 0.5  µg/mL (1), which would not completely eliminate the misclassification as susceptible. Furthermore, WHO endorsed a composite reference standard, whereby resistance is defined as MICs > CC (i.e., phenotypic resistance) plus strains with any mutation listed in the latest edition of the WHO mutation catalog (28). In other words, strains that are phenotypically susceptible but have a borderline resistance mutation are considered resistant (36).

The post-docking analysis revealed the binding patterns of RIF with the WT and four borderline mutation models (L452P, L430P, and H445N) in terms of docking scores, H-bond formation, and their associated distances. Furthermore, the MD analysis showed that the four borderline RIF-resistance patterns detected in our study did not affect the binding of the protein and RIF, even increasing the affinity with lower ΔG_binding_ values compared with the WT rpoB. The present findings showed that the three borderline RIF resistance-associated mutations (L452P, L430P, and H445N) may not affect the binding affinity of rpoB and RIF, which hinted that the resistance may be caused by other mechanisms. Thus, the mechanism of borderline RIF resistance-associated mutations needs further investigation.

Our study has some limitations. Firstly, WHO lowered the MGIT critical concentration to 0.5 µg/mL in 2021, and the recommended critical concentration of RIF by the manufacturer’s instructions in China was still 1 µg/mL during our study period (January 2020 to December 2022), which caused more false discordant results between Xpert and MGIT960 regarding RIF susceptibility. Secondly, we only focused on cases that were determined to be MTB positive by the Xpert assay, rather than on all cases detected by MGIT960 or Xpert assay with discordant RIF-susceptible results. Thirdly, the Mtb clinical isolates were obtained from a single hospital, Wenzhou Central Hospital, located in the south-east of China. Thus, many of the isolates may be related genetically and epidemiologically and more clinical isolates are needed to assess the borderline RIF resistance in settings with different epidemiological backgrounds.

In conclusion, the present findings can help interpret false-positive resistance to RIF by Xpert, which is probably caused by borderline RIF resistance-associated mutations. Additionally, three borderline RIF resistance-associated mutations (L430P, H445N, and L452P) may not affect the binding affinity between *rpoB* and RIF and the corresponding resistant mechanism needs further investigation. Moreover, DNA sequencing of *rpoB* was recommended to evaluate the association with resistance to RIF for discordant RIF susceptibility test results.

## Data Availability

All data relevant to this study are supplied in the manuscript and supplementary files or are available from the corresponding author upon request. The whole genome sequencing data of 28 strains were submitted to the NCBI (PRJNA1079416: *Mycobacterium tuberculosis*).

## SUPPLEMENTAL MATERIAL

Figure S1 spectrum.03859-23-s0001.tif Post-docking analysis: 2D binding modes of RIF.

Table S1 spectrum.03859-23-s0002.docx Docking scores and post-docking interactions.

ASM does not own the copyrights to Supplemental Material that may be linked to, or accessed through, an article. The authors have granted ASM a non-exclusive, world-wide license to publish the Supplemental Material files. Please contact the corresponding author directly for reuse.
