# Supplementary figures and images for: Discordant results between Xpert MTB/RIF assay and Bactec MGIT 960 culture system regarding the detection of rifampin-resistant Mycobacterium tuberculosis isolates in Wenzhou, China
Source: Microbiol Spectr. 2024 May 13;12(6):e03859-23. doi: 10.1128/spectrum.03859-23 (PMC11237732; doi:10.1128/spectrum.03859-23)

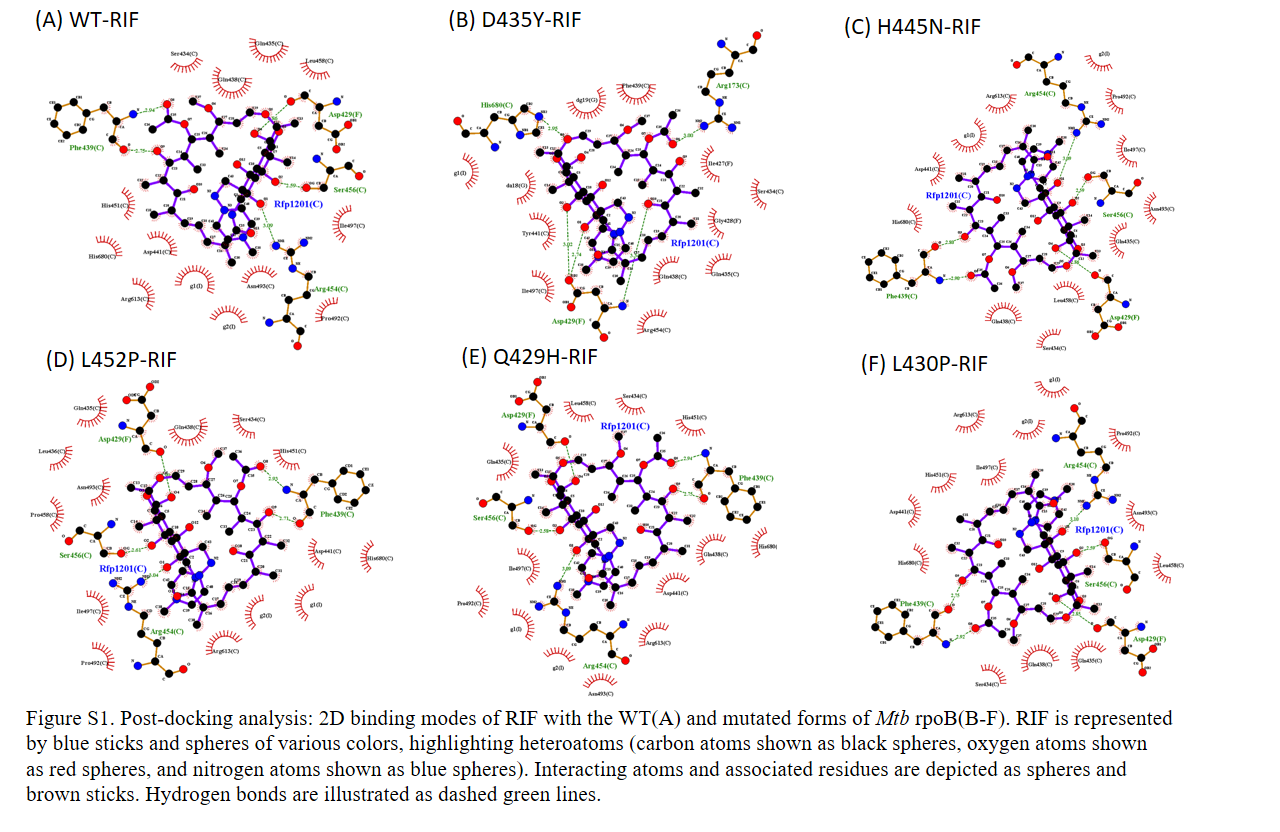

Supplement: Figure S1 — Post-docking analysis: 2D binding modes of RIF. [file spectrum.03859-23-s0001.tif]
