# Supplementary material for: Discordant results between Xpert MTB/RIF assay and Bactec MGIT 960 culture system regarding the detection of rifampin-resistant Mycobacterium tuberculosis isolates in Wenzhou, China
Source: Microbiol Spectr. 2024 May 13;12(6):e03859-23. doi: 10.1128/spectrum.03859-23 (PMC11237732; doi:10.1128/spectrum.03859-23)
Supplement: Table S1 — Docking scores and post-docking interactions. [file spectrum.03859-23-s0002.docx]

Table S1. Docking scores and post-docking interactions (hydrogen bonds) of RIF in WT and mutated models of rpoB.
